# Effect of Omega-3 Polyunsaturated Fatty Acids on Cardiovascular Outcomes in Patients with Diabetes: A Meta-analysis of Randomized Controlled Trials

**DOI:** 10.1016/j.advnut.2023.04.009

**Published:** 2023-04-28

**Authors:** Linlin Huang, Fanjie Zhang, Ping Xu, Yijie Zhou, Yijun Liu, Hongdie Zhang, Xiaozhen Tan, Xinxu Ge, Yong Xu, Man Guo, Yang Long

**Affiliations:** 1 Experimental Medicine Center, the Affiliated Hospital of Southwest Medical University, Luzhou, Sichuan, China; 2 Department of Endocrinology and Metabolism, the Affiliated Hospital of Southwest Medical University, Luzhou, Sichuan, China; 3 Metabolic Vascular Disease Key Laboratory of Sichuan Province, Luzhou, Sichuan, China; 4 Academician (Expert) Workstation of Sichuan Province, the Affiliated Hospital of Southwest Medical University, Luzhou, Sichuan, China

**Keywords:** cardiovascular disease, docosahexaenoic acid, eicosapentaenoic acid, meta-analysis, omega-3 polyunsaturated fatty acids, diabetes

## Abstract

The current guidelines recommend that people consume 2 or more servings of fat-rich fish per week to obtain enough omega-3 (ω-3) polyunsaturated fatty acids to prevent cardiovascular events. However, the cardiovascular benefits of ω-3 polyunsaturated fatty acids in patients with diabetes are unclear, and related large-scale trials have produced conflicting results. We aimed to perform a meta-analysis of all randomized controlled trials that attempted to assess the effects of ω-3 fatty acid supplementation on cardiovascular outcomes in patients with diabetes. In PubMed, EMBASE, and the Cochrane Library, we searched for data from all randomized controlled trials on ω-3 fatty acids and cardiovascular outcomes in patients with diabetes published before July 2022. Eight eligible studies involving 57,754 participants were ultimately included. Meta-analysis showed that ω-3 fatty acid supplementation reduces cardiovascular disease (CVD) risk in patients with diabetes (rate ration [RR] = 0.93; 95% confidence interval [CI]: 0.90, 0.97; *P* = 0.0009). Among them, eicosapentaenoic acid (EPA), but not EPA plus docosahexaenoic acid (DHA), significantly reduced the risk of CVD in patients with diabetes (EPA [RR = 0.81; 95% CI: 0.73, 0.90; *P*=0.0001]). This meta-analysis suggests that ω-3 fatty acid supplementation is an effective strategy to prevent CVD in patients with diabetes, but further well-designed, large-scale randomized controlled trials are necessary to evaluate the safety of ω-3 fatty acid supplementation, and its effect on atrial fibrillation. This study was registered with PROSPERO as CRD42022346302.

**Statements of significance**

This study found that supplementation with omega-3 fatty acids reduced the risk of cardiovascular disease in patients with diabetes. Among them, eicosapentaenoic acid but not eicosapentaenoic acid plus docosahexaenoic acid, significantly reduced the risk of cardiovascular disease in patients with diabetes.

## Introduction

Omega-3 (ω-3) PUFAs mainly include ALA, EPA, DPA, and DHA, of which EPA and DHA are derived from the lipids of fatty fish, the liver of white lean fish, and the fat of marine mammals and have received the most attention from researchers. DPA is found in less concentration in fish oils, whereas ALA is mainly present in vegetable oils, such as those derived from seeds and nuts [1]. Omega-3 FAs are an important part of a heart-healthy diet. CVD is a major contributor to death in people with diabetes and is a key target of diabetes care [2,3]. A recent meta-analysis of randomized controlled trials (RCTs) showed that ω-3 FA supplementation has a hypolipidemic effect in patients with type 2 diabetes [4]. However, the effect of ω-3 FAs on the risk of CVD in patients with diabetes has been controversial. The Gruppo Italiano per lo Studio della Sopravvivenza nell'Infarto miocardico (GISSI)-heart failure trial, an RCT involving 1974 participants with diabetes, showed that patients supplemented with ω-3 FAs had an 11% lower risk of hospitalization for CVD and all-cause mortality than placebo controls [5]. The Reduction of Cardiovascular Events with Icosapent Ethyl-Intervention Trial (REDUCE-IT), a trial studying a highly purified EPA ethyl ester icosapent ethyl, found a 23% reduction in the risk of major cardiovascular events in participants with diabetes in the icosapent ethyl group [6]. In addition, the Vitamin D and Omega-3 Trial (VITAL) recently reported a 31% reduction in initial heart failure hospitalizations and a 47% reduction in recurrent heart failure hospitalizations in patients with type 2 diabetes who were supplemented with ω-3 FAs [7]. However, the A Study of Cardiovascular Events in Diabetes (ASCEND) trial reported that supplementation with ω-3 FAs did not significantly reduce cardiovascular events in patients with diabetes compared with the placebo group [8]. Studies have also found that supplementation with ω-3 FAs increases the risk of atrial fibrillation in patients with diabetes.

Therefore, it is unclear whether ω-3 FA supplementation reduces the risk of CVD in patients with diabetes, and the reasons for the inconsistent results of RCTs are unknown. The purpose of this review was to conduct a meta-analysis of RCTs to provide a comprehensive and up-to-date assessment of the effects of ω-3 FA consumption on cardiovascular outcomes in patients with diabetes mellitus, to explore the factors influencing the effect of ω-3 FA supplementation on diabetic CVD, and to identify appropriate interventions in which ω-3 FA supplementation may have the greatest therapeutic effect.

## Methods

The study protocol is prospectively registered in PROSPERO database (no. CRD42022346302). This study follows the PRISMA guidelines [9].

### Data sources and searches

We systematically searched the PubMed, EMBASE, and Cochrane Library databases for all data published before July 2022. Medical subject headings and free-text terms were combined to retrieve relevant articles without any language restrictions. Details of the search terms are shown in Supplemental Table S1. In addition, we manually searched the references in the selected trials and reviews to ensure that all relevant articles were included in the search. Selection criteria were developed by 2 reviewers (LLH and FJZ), and after excluding duplicate articles, relevant articles were screened based on the titles and abstracts of the retrieved articles. Subsequently, 2 authors (LLH and PX) independently assessed the full texts of potentially suitable articles to determine their eligibility for inclusion according to the predetermined criteria. Any disagreements that arose were decided by the reviewers through discussion or by a third reviewer.

### Study selection

Participants with diabetes, including but not limited to type 1 diabetes and type 2 diabetes, were included in this meta-analysis. We included studies for analysis that met the following criteria: *1*) RCTs in which participants were aged ≥18 y of with diabetes; 2) all ω-3 FA interventions were administered in dietary or capsule form, and the dose and timing could be determined; and *3*) the incidence of CVD associated with different ω-3 FAs was reported. Observational studies and RCTs involving children or pregnant women were excluded. This review focused on the following outcomes: cardiovascular events (cardiovascular death or hospitalization for cardiovascular causes, fatal and/or nonfatal MI, angina, fatal and/or nonfatal stroke, heart failure, unplanned revascularization, and atrial fibrillation) and all-cause mortality (Table 1).

**TABLE 1:** Details of the selected studies and baseline characteristics of the participants

Study	Y	Patients, no.	Mean age, y	Male, no. (%)	Median followup duration, y	Basic CVD	Intervention arm	Major clinical outcomes
JELIS [12]	2007	3040	61	NA	4.6	With or without CAD (previous MI, coronary, interventions, or confirmed angina pectoris)	1.8 g/d EPA	Major coronary events (sudden cardiac death, fatal and nonfatal MI, and other nonfatal events, including unstable angina pectoris, angioplasty, stenting, or coronary artery bypass grafting)
GISSI-HF [5]	2008	1974	67	NA	3.9	Chronic heart failure	1 g/d of ω-3 Fas (850–882 mg EPA and DHA as ethyl esters in the average ratio of 1:1.2)	All-cause death or admission to hospital for cardiovascular reasons
ORIGINALE [13]	2012	12536	64	65	6.2	A history of MI, stroke, or revascularization; angina with documented ischemia; a ratio of urinary albumin to creatinine of >30 mg per gram; left ventricular hypertrophy; ≥50% stenosis of a coronary, carotid, or lower-limb artery on angiography; or an ankle-brachial index of <0.9	1 g/d of ω-3 FAs (465 mg EPA and 375 mg DHA)	Death from cardiovascular causes
Risk and prevention [14]	2013	7494	64	NA	5	1 CVD risk factor	1 g/d of ω-3 FAs (EPA and DHA content not <85%, in a ratio that could range from 0.9:1–1.5:1)	The time to death from cardiovascular causes or first hospital admission for cardiovascular causes
ASCEND [8]	2018	15,480	63.3	62.6	7.4	No	1 g/d ω-3 FAs (460 mg EPA and 380 mg DHA)	Nonfatal MI, nonfatal ischemic stroke, transient ischemic attack、vascular death
REDUCE-IT [6]	2019	4787	64	NA	4.9	≥1 additional CVD risk factor	4 g/d Icosapent ethyl (ethyl ester of EPA)	Cardiovascular death, nonfatal MI (including silent MI), nonfatal stroke, coronary revascularization, or unstable angina
VITAL [10]	2019	3549	67.1	NA	5.3	No	1 g/d ω-3 FAs (460 mg EPA and 380 mg DHA)	MI, stroke, and cardiovascular mortality
STRENGTH [15]	2020	9170	62.5	NA	3.5	≥1 additional CVD risk factor	4 g/d ω-3 FAs (EPA and DHA)	Cardiovascular death, nonfatal MI, nonfatal stroke, coronary revascularization, and hospitalization for unstable angina
VITAL [11]	2021	3442	66.7	NA	5.3	No	1 g/d ω-3 FAs (460 mg EPA and 380 mg DHA)	Atrial fibrillation
VITAL-HF [7]	2022	3537	67.1	49.4	5.3	No	1 g/d ω-3 FAs (460 mg EPA and 380 mg DHA)	The first hospitalization for heart failure

ASCEND, A Study of Cardiovascular Events in Diabetes; GISSI, the Gruppo Italiano per lo Studio della Sopravvivenza nell'Infarto miocardico; JELIS, Japan EPA Lipid Intervention Study; REDUCE-IT, the Reduction of Cardiovascular Events with Icosapent Ethyl-Intervention Trial; VITAL, Vitamin D and Omega-3 Trial.

### Data extraction and quality assessment

A standardized data extraction form was developed and included the following information: trial name, year and country of publication, study design, the number of participants included in the analysis, age and gender of participants, underlying CVD of participants, the content of the intervention and control, the duration of the intervention, and primary outcome of the study. Data were extracted independently by the first author, checked for accuracy by the second author, and discussed and decided by all authors in case of discrepancies. Two researchers independently assessed the quality of the study using the Cochrane tool for assessing risk of bias based on the following: random sequence generation, allocation concealment, blinding of participants, personnel and outcome assessment, incomplete outcome data, selective outcome reporting, and other biases. Each RCT was assigned 1 of 3 levels of “high risk,” “low risk,” and “unclear risk,” and details of the risk of bias assessment are provided in Supplemental Figure S1.

### Data synthesis and analysis

We used Cochrane’s Review Manager version 5.3 (the Cochrane Collaboration) and Stata version 17.0 (Stata) to analyze all data and a *P* value of <0.05 indicated statistical significance unless otherwise stated. We used the pooled rate ratio (RR) and 95% CI to evaluate the results. We used *I*^2^ to assess the heterogeneity among the included studies. A fixed effect model was used when the *I*^2^ value was ≤50% and a random effects model was used when the *I*^2^ statistic was >50%. Sensitivity analyses were performed by deleting each study in turn and performing a new meta-analysis on the remaining data. Publication bias was assessed using funnel plots and Egger’s test. Moreover, we also performed subgroup analyses according to an ethnic group, type and dose of ω-3 FA supplementation, and different cardiovascular outcomes to explore the reasons for the differences between the studies.

## Results

### Literature search and study characteristics

**FIGURE 1:**
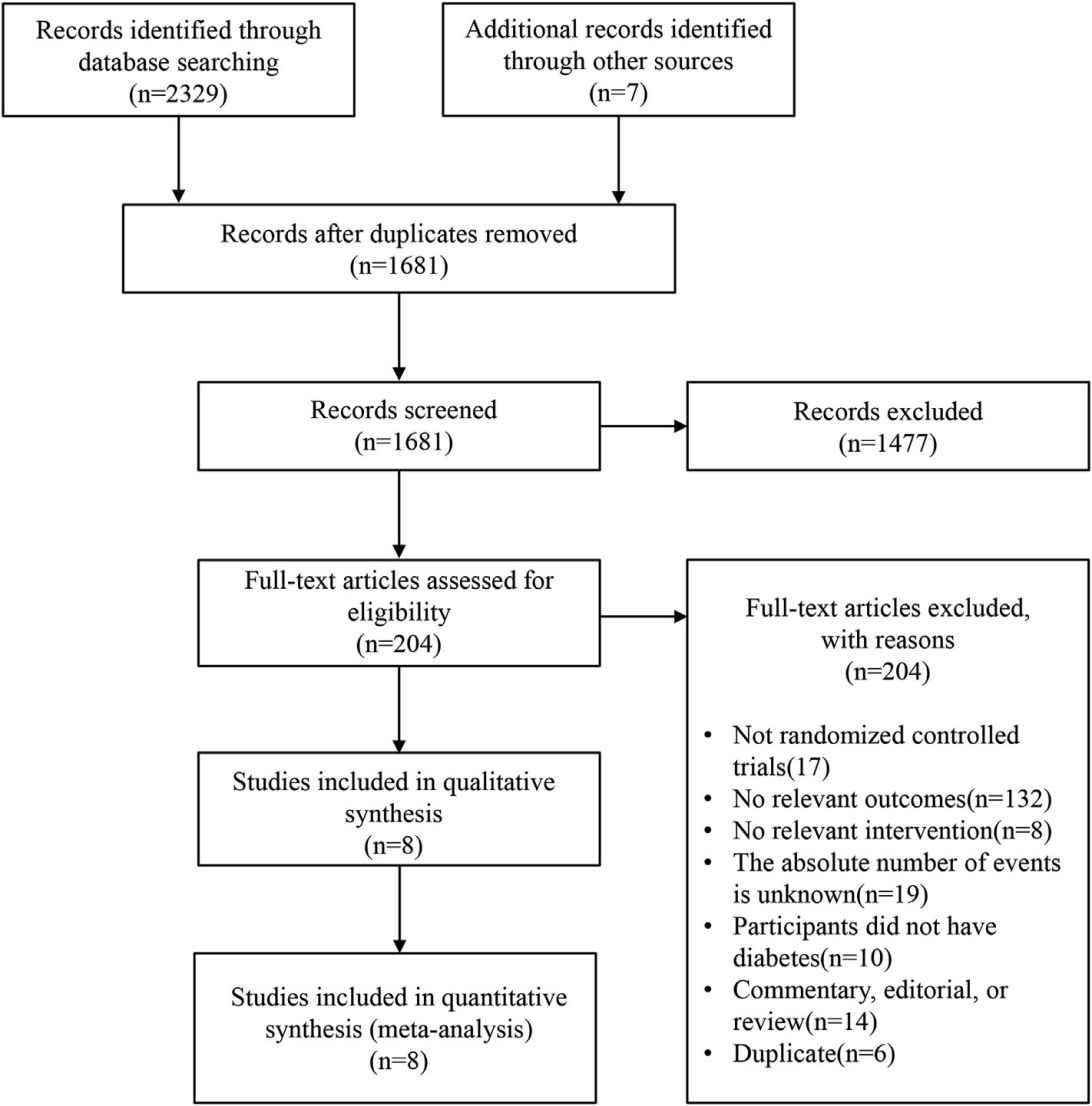
Flow chart of the literature search and study selection process.

An initial search identified 2336 relevant studies, and after excluding 655 duplicate reports, their titles and abstracts were screened according to the above criteria. We then included 8 of the 204 full-text reports evaluated in the meta-analysis, the process of which is shown in Figure 1. Although the 3 studies of the VITAL trial focused on different cardiovascular outcomes, the interventions and study populations were the same [7,10,11]. Therefore, we selected the VITAL-heart failure (VITAL-HF) trial with the largest study population for the primary analysis [7]). A total of 57,754 patients were included in the 8 studies [[5], [6], [7], [8],[12], [13], [14], [15]] with 28,906 patients in the ω-3 FA supplementation group and 28,848 patients in the control group, and the detailed basic characteristics of each study are shown in Table 1. These articles reported different types of ω-3 FA supplementation. Six studies tested EPA plus DHA at doses ranging from 850 to 4000 mg/d [5,7,8,13,14,15], whereas the Japan EPA Lipid Intervention Study (JELIS) and REDUCE-IT trials tested EPA alone at doses ranging from 1800 to 4000 mg/d [6,12]. Only the VITAL-HF trial explicitly included patients with type 2 diabetes, and the remaining trials did not specify the type of diabetes [7]. All studies were double-blind, placebo-controlled trials, except for the JELIS trial [12], which was an open-label trial. These clinical trials were conducted in different countries around the world: 2 from Italy [5,14], 1 from the United States [7], 1 from the United Kingdom [8], 1 from Japan [12], and 3 international multicenter trials [6,13,15].

#### Omega-3 FA supplementation significantly reduced CVD in patients with diabetes

**FIGURE 2:**
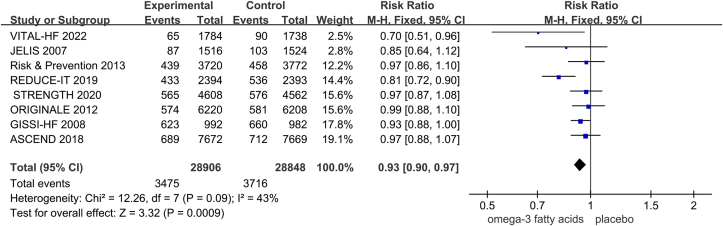
Pooled association between ω-3 FA supplementation and risk of CVD in participants with diabetes.

For the primary composite cardiovascular outcome, with 8 studies involving 57,754 patients, the number of events was 3475 in the ω-3 FA supplementation group and 3716 in the control group. Fixed-effects model analysis showed that ω-3 FA supplementation significantly reduced cardiovascular events in patients with diabetes (RR = 0.93; 95% CI: 0.90, 0.97; *P* = 0.0009; *I*^2^=43%; Figure 2). To improve the accuracy of the results, we included the remaining 2 studies from the VITAL trial in another 2 meta-analyses and found that the results of both studies were consistent with the primary analysis (Supplemental Figures S2 and S3). To investigate the effect of ω-3 FA supplementation on the risk of different CVDs, we performed a series of subgroup analyses and found no significant association between ω-3 FA supplementation and the risk of total MI (fatal and nonfatal MI), major vascular events, CAD, atrial fibrillation, and all-cause mortality in participants with diabetes (Supplemental Figures S4–S8).

#### EPA alone was more effective in reducing the risk of CVD in patients with diabetes than EPA combined with DHA

**FIGURE 3:**
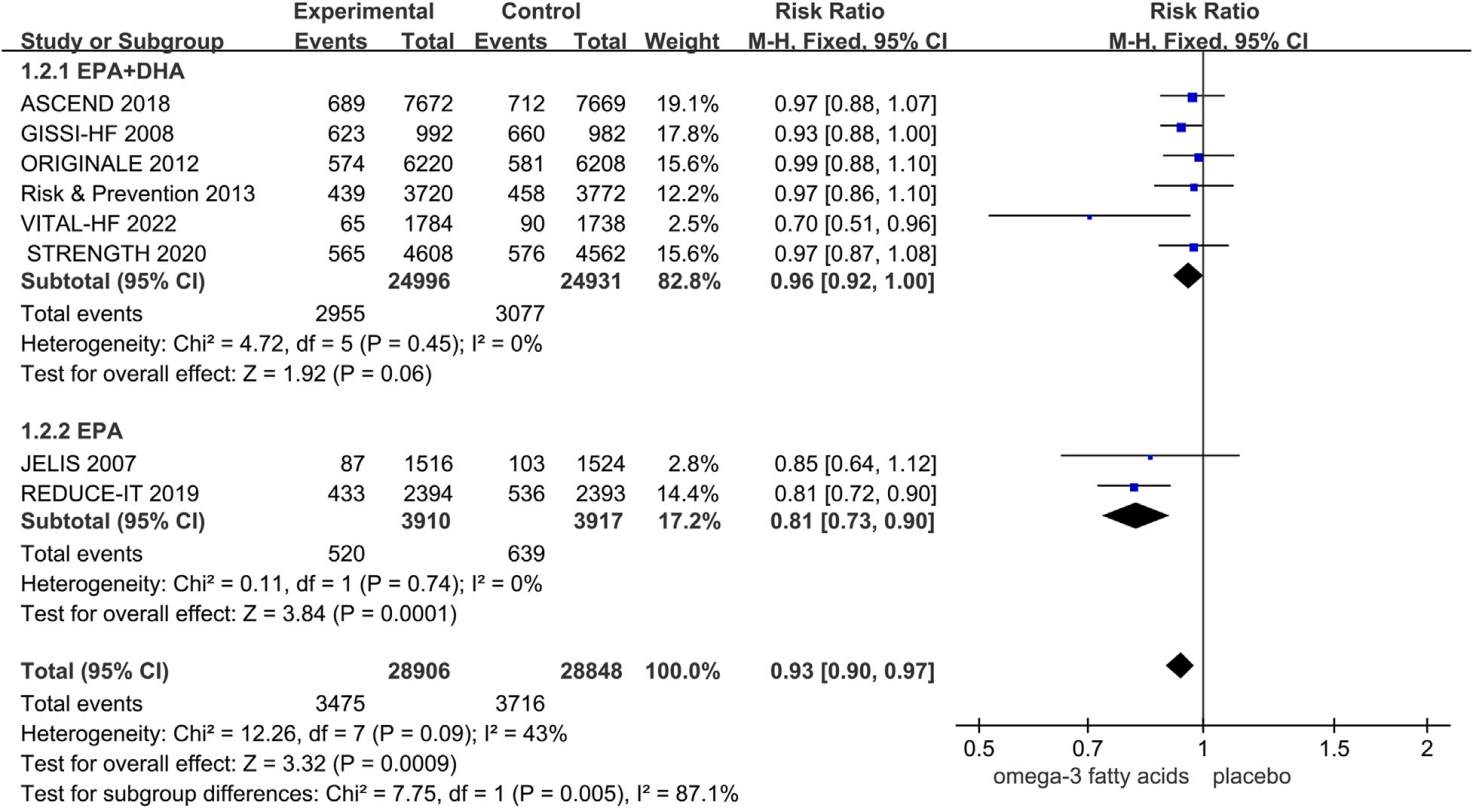
Pooled associations between different types of ω-3 FA supplementation and cardiovascular outcomes in diabetes.

To further investigate the effects of different types of ω-3 FA supplementation on CVD in patients with diabetes, we conducted a subgroup analysis of 8 studies involving 57,754 participants. Six studies involving 49,927 participants reported on the effect of EPA combined with DHA on the risk of CVD in patients with diabetes. Two studies involving 7827 participants reported on the effect of EPA alone on the risk of CVD in patients with diabetes. EPA alone significantly reduced the risk of CVD in patients with diabetes, whereas the combination of EPA and DHA was not significantly associated with it (EPA [RR = 0.81; 95% CI: 0.73, 0.90; *P* = 0.0001; *I*^2^=0%]; Figure 3).

### Sensitivity analyses

**FIGURE 4:**
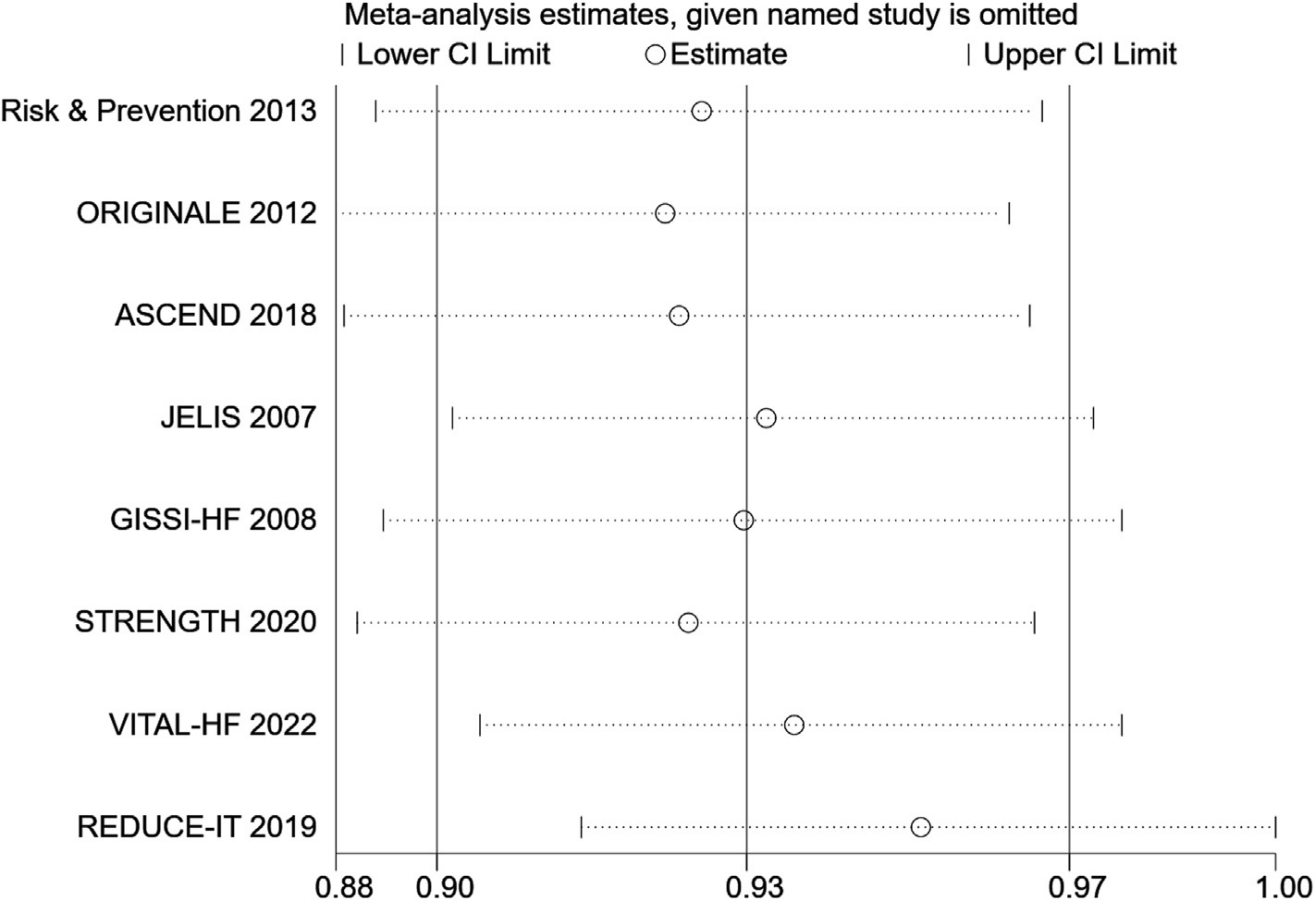
Sensitivity analysis of CVD.

A sensitivity analysis comparing cardiovascular outcomes was performed by removing 1 study at a time and observing its effect on overall outcomes. The point estimates after excluding each study were within the 95% CI of the total effect size, indicating that the results of this meta-analysis are stable and reliable (Figure 4). Sensitivity analysis showed that excluding any of the studies did not affect the overall results. However, in the subgroup analysis based on different types of ω-3 FA supplementation, a significant reduction in study heterogeneity was found, suggesting that different types of ω-3 FA supplementation are a source of study heterogeneity (Figure 3).

## Publication bias

We assessed publication bias for cardiovascular outcomes using funnel plots (Supplemental Figure S9) and Egger’s test (*P* = 0.283; 95% CI: −4.217702, 1.47373, Supplemental Figure S10) and found no evidence of publication bias.

## Discussion

This meta-analysis of 8 studies (57,754 patients) examined the effects of ω-3 FA supplementation on cardiovascular outcomes in patients with diabetes. We extracted multiple cardiovascular events occurring in patients with diabetes from these studies and performed several statistical analyses to assess the effect of ω-3 FA supplementation on the risk of CVD in patients with diabetes. We found that ω-3 FA supplementation significantly reduced the risk of CVD in patients with diabetes. However, subgroup analysis found that EPA alone, but not EPA plus DHA, significantly reduced the risk of CVD in participants with diabetes. These data suggest that supplementation with ω-3 FAs, particularly EPA, significantly reduces the risk of CVD in patients with diabetes.

Emerging evidence shows that the consumption of ω-3 FAs improves some risk factors for CVD. A recent meta-analysis showed that ω-3 supplementation significantly reduced cardiometabolic biomarkers, such as LDL, very LDL, and TGs in patients with type 2 diabetes [4]. A recent systematic review of clinical trials has shown that supplementation with ω-3 FAs reduces advanced glycation end products, which are associated with increased cardiovascular events in patients with type 2 diabetes [16,17]. Moreover, ω-3 FAs have protective effects on pathophysiologic processes, such as inflammation, and endothelial dysfunction, in diabetes. An RCT by Tousoulis et al. [18] showed that ω-3 FAs improved endothelial function and arterial stiffness in patients with metabolic syndrome and had anti-inflammatory effects. Animal studies have shown that EPA and DHA reduce atherosclerosis in mice by inhibiting the activation of macrophages [19]. In addition, ω-3 FAs regulate the function of T cells and produce the antithrombotic metabolites thromboxane A3, prostacyclin, and specialized proresolving lipid mediators, such as resolvins, maresins, and protectins, which promote tissue repair and inflammation, and play an important role in the inflammatory mechanisms of atherosclerosis [20]. Therefore, ω-3 FA supplementation may be a potential therapeutic approach to reduce the risk of CVD in patients with diabetes.

As previously mentioned, EPA alone significantly reduced the risk of CVD in participants with diabetes, whereas EPA plus DHA may reduce the risk of CVD in participants with diabetes but not significantly. It has been shown that DHA increases LDL concentrations, whereas EPA does the opposite [21,22]. In vitro experiments reported that combined treatment with EPA and statins improved endothelial dysfunction induced by hyperglycemia and oxidative stress, whereas DHA did not exert a similar effect [23]. Furthermore, unlike DHA, EPA can enhance the atherosclerotic protective function of HDL by inhibiting the oxidation of HDL particles [24]. However, the doses of EPA used in the REDUCE-IT and JELIS trials were higher than the doses of DHA plus EPA used in trials other than the STRENGTH trial [6,12,15]. A recent meta-analysis showed that higher doses of ω-3 FA supplementation may increase their protective effect against CVD [25]. Therefore, we are not yet sure that the use of EPA alone is more advantageous than the use of EPA plus DHA for the prevention of CVD in patients with diabetes, and further intervention trials controlling for other variables, such as dose, are needed to confirm.

Two studies from the VITAL trial showed greater cardiovascular benefits of ω-3 FA supplementation in Blacks with diabetes than in Whites [10,11]. Consistently, an observational study found racial differences in the association between ω-3 biomarkers and coronary events and also a stronger correlation in African Americans [26]. Defining the role of genetic factors may be the key to understanding the stronger protective effect of ω-3 FA supplementation on diabetic CVD in Black individuals. It has been reported that the fatty acid desaturase 1 (FADS1) and FADS2 genes play an important role in the adaptation of the Inuit diet and are associated with coronary artery disease, and the variants in these genes may affect the biosynthesis of ω-3 FAs [27,28]. In addition, patients of European descent with diabetes or metabolic syndrome differ from those of African descent concerning FADS variants [29,30]. Differences in dietary, clinical, and environmental factors between races may have contributed to this result [31,32].

However, the use of ω-3 FA supplements for the prevention of CVD in diabetes should be accompanied by a consideration of their safety. In addition to their protective effect against diabetic CVD, ω-3 FA supplementation may increase the risk of atrial fibrillation in patients with diabetes, but not significantly. The Omega-3 Fatty Acids in the Elderly with Myocardial Infarction (OMEMI) trial also reported that ω-3 FA supplements may be associated with a nonsignificant increased risk of atrial fibrillation in older patients with diabetes who have had a MI [33]. A recent meta-analysis showed that supplementation with ω-3 FAs was significantly associated with an increased risk of atrial fibrillation, and the risk appeared to be increased at doses >1 g/d [34]. Because the benefits of ω-3 FA supplementation also appear to be dose dependent, the risks associated with atrial fibrillation should be balanced against the benefits of CVD.

### Study limitations

Our study has several major limitations. First, most of the included trials studied patients with CVD risk factors, including diabetes, and there were differences in patient comorbidities and inclusion criteria. The ASCEND trial enrolled a population that did not include patients with CVD. However, the population included in the GISSI-heart failure trial was patients with heart failure. Second, only the VITAL-HF trial studied the cardiovascular effect of ω-3 in patients with type 2 diabetes, and the remaining trials did not specify the type of diabetes. Therefore, we reported the beneficial effects of ω-3 supplementation in patients with diabetes. Further studies are warranted to determine the effect of ω-3 on cardiovascular outcomes in different types of diabetes. Third, most of the study participants were from North American and European countries, limiting the applicability of the findings to Asian populations or others. Fourth, most of the included studies reported only the total number of cardiovascular events in the diabetic population, limiting our subgroup analysis of the various types of cardiovascular events. Finally, because the treatment effects between subgroups were analyzed according to a single factor, we cannot yet clarify whether EPA alone is more effective in reducing the risk of CVD in patients with diabetes compared with the combination of DHA by excluding the effect of dose.

## Conclusion

Omega-3 FA supplementation is an effective strategy to prevent cardiovascular outcomes in patients with diabetes. EPA alone was more effective than the combined supplementation of DHA and EPA. However, the effect of dose cannot yet be ruled out, and larger trials controlling for dose and other variables are needed to confirm this. Moreover, the safety of ω-3 FA supplements should be considered in their clinical applications. An increased risk of atrial fibrillation with ω-3 FA supplementation in patients with diabetes cannot be ruled out at this time, and this may have clinical implications that need to be verified in larger, comparative-dose clinical trials.

## Acknowledgments

The authors’ responsibilities were as follows – LLH, YL, MG: were involved in the conception and design of the study; LLH, FJZ, PX: performed the systematic literature search, extracted the data, and quality assessment; LLH, FJZ, PX, YJZ: analyzed the data and drafted the manuscript; YJZ, YJL, HDZ, XZT: revised the manuscript; LLH, FJZ, XXG, YX: performed data interpretation and revised the manuscript; YL, MG: reviewed the manuscript and assumed primary responsibility for the final content. All authors: read and agreed to the published version of the manuscript.

### Funding

Supported by the National Natural Science Foundation of China (No. 82171860), the Department of Science and Technology of Sichuan Province (No.22ZDYF3804), the Health Commission of Sichuan Province (No. 21PJ097), and the Luzhou-Southwest Medical University cooperation project (No. 2019LZXNYDJ35 and No. 2021LZXNYD-D09).

### Availability of data and materials

All data generated or analyzed during this study are included in this published article and its supplementary information files.

### Author disclosures

The authors report no conflicts of interest.
