# Supplementary material for: Effect of Omega-3 Polyunsaturated Fatty Acids on Cardiovascular Outcomes in Patients with Diabetes: A Meta-analysis of Randomized Controlled Trials
Source: Adv Nutr. 2023 Apr 28;14(4):629–36. doi: 10.1016/j.advnut.2023.04.009 (PMC10334152; doi:10.1016/j.advnut.2023.04.009)
Supplement: Multimedia component 1 [file mmc1.docx]

**Table S1.** Search strategy

| **Data source** | **Search terms** |
| --- | --- |
| PubMed | #1 Diabetes Mellitus [MeSH Terms]  #2 diabetes OR diabetic OR diabetics [Title/Abstract]  #3 #1 OR #2  #4 Fatty Acids, Omega-3 [MeSH Terms]  #5 Omega-3 Fatty Acid OR Acid, Omega-3 Fatty OR Fatty Acid, Omega-3 OR Omega 3 Fatty Acid OR Omega-3 Fatty Acids OR n-3 Oil OR Oil, n-3 OR n 3 Oil OR n3 Oil OR Oil, n3 OR n-3 Fatty Acids OR n 3 Fatty Acids OR Omega 3 Fatty Acids OR n-3 PUFA OR PUFA, n-3 OR n 3 PUFA OR n3 Fatty Acid OR Fatty Acid, n3 OR n3 PUFA OR PUFA, n3 OR n3 Polyunsaturated Fatty Acid OR n3 Oils OR n-3 Oils OR n 3 Oils OR N-3 Fatty Acid OR Acid, N-3 Fatty OR Fatty Acid, N-3 OR N 3 Fatty Acid OR n-3 Polyunsaturated Fatty Acid OR n 3 Polyunsaturated Fatty Acid OR EPA OR eicosapentaenoic OR DHA OR alpha-linolenic acid OR ALA OR docosahexaenoic OR fish OR marine[Title/Abstract]  #6 #4 OR #5  #7 Cardiovascular Diseases [MeSH Terms]  #8 Cardiovascular Disease OR Disease, Cardiovascular OR Diseases, Cardiovascular OR heart failure OR Myocardial Infarction OR Atrial fibrillation OR cardiovascular death OR cardiovascular mortality OR coronary artery disease OR stroke [Title/Abstract]  #9 #7 OR #8  #10 randomized controlled trial OR controlled clinical trial [Publication Type]  #11 randomized OR placebo OR randomly OR trial OR groups [Title/Abstract]  #12 #10 OR #11  #13 #3 AND #6 AND #9 AND #12 |
| EMBASE | ('Diabetes mellitus' /exp OR 'diabetes': ab,ti OR 'diabetic':ab,ti OR 'diabetics':ab,ti ) AND ('Omega 3 fatty acids'/exp OR 'Omega-3 Fatty Acid': ab,ti OR 'Acid, Omega-3 Fatty':ab,ti OR 'Fatty Acid, Omega-3':ab,ti OR 'Omega 3 Fatty Acid':ab,ti OR 'Omega-3 Fatty Acids':ab,ti OR 'n-3 Oil':ab,ti OR 'Oil,n-3':ab,ti OR 'n 3 Oil':ab,ti OR 'n3 Oil':ab,ti OR 'Oil, n3':ab,ti OR 'n-3 Fatty Acids':ab,ti OR 'n 3 Fatty Acids':ab,ti OR 'Omega 3 Fatty Acids':ab,ti OR 'n-3 PUFA':ab,ti OR 'PUFA, n-3':ab,ti OR 'n 3 PUFA':ab,ti OR 'n3 Fatty Acid':ab,ti OR 'Fatty Acid, n3':ab,ti OR 'n3 PUFA':ab,ti OR 'PUFA, n3':ab,ti OR 'n3 Polyunsaturated Fatty Acid':ab,ti OR 'n3 Oils':ab,ti OR 'n-3 Oils':ab,ti OR 'n 3 Oils':ab,ti OR 'N-3 Fatty Acid':ab,ti OR 'Acid, N-3 Fatty':ab,ti OR 'Fatty Acid, N-3':ab,ti OR 'N 3 Fatty Acid':ab,ti OR 'n-3 Polyunsaturated Fatty Acid ':ab,ti OR 'n 3 Polyunsaturated Fatty Acid':ab,ti OR 'EPA':ab,ti OR 'eicosapentaenoic':ab,ti OR 'DHA':ab,ti OR 'alpha-linolenic acid':ab,ti OR 'ALA':ab,ti OR 'docosahexaenoic':ab,ti OR 'fish':ab,ti OR 'marine':ab,ti) AND ('Cardiovascular Diseases'/exp OR 'Cardiovascular Disease': ab,ti OR 'Disease, Cardiovascular': ab,ti OR 'Diseases, Cardiovascular': ab,ti OR 'heart failure': ab,ti OR 'Myocardial Infarction': ab,ti OR 'Atrial fibrillation': ab,ti OR 'cardiovascular death': ab,ti OR 'cardiovascular mortality': ab,ti OR 'coronary artery disease': ab,ti OR 'stroke': ab,ti) AND ('randomized controlled trial':it OR 'controlled clinical trial':it OR 'randomized': ab,ti OR 'placebo': ab,ti OR 'randomly': ab,ti OR 'trial': ab,ti OR 'groups': ab,ti) |
| Cochrane Library | #1 MeSH descriptor: [Cardiovascular Diseases] explode all trees  #2 (diabetes OR diabetic OR diabetics):ti,ab,kw  #3 #1 OR #2  #4 MeSH descriptor: [Fatty Acids, Omega-3] explode all trees  #5 (Omega-3 Fatty Acid OR Acid, Omega-3 Fatty OR Fatty Acid, Omega-3 OR Omega 3 Fatty Acid OR Omega-3 Fatty Acids OR n-3 Oil OR Oil, n-3 OR n 3 Oil OR n3 Oil OR Oil, n3 OR n-3 Fatty Acids OR n 3 Fatty Acids OR Omega 3 Fatty Acids OR n-3 PUFA OR PUFA, n-3 OR n 3 PUFA OR n3 Fatty Acid OR Fatty Acid, n3 OR n3 PUFA OR PUFA, n3 OR n3 Polyunsaturated Fatty Acid OR n3 Oils OR n-3 Oils OR n 3 Oils OR N-3 Fatty Acid OR Acid, N-3 Fatty OR Fatty Acid, N-3 OR N 3 Fatty Acid OR n-3 Polyunsaturated Fatty Acid OR n 3 Polyunsaturated Fatty Acid OR EPA OR eicosapentaenoic OR DHA OR alpha-linolenic acid OR ALA OR docosahexaenoic OR fish OR marine) :ti,ab,kw  #6 #4 OR #5  #7 MeSH descriptor: [Cardiovascular Diseases] explode all trees |
|  | #8 (Cardiovascular Disease OR Disease, Cardiovascular OR Diseases, Cardiovascular OR heart failure OR Myocardial Infarction OR Atrial fibrillation OR cardiovascular death OR cardiovascular mortality OR coronary artery disease OR stroke) :ti,ab,kw  #9 #7 OR #8  #10 #3 AND #6 AND #9 |


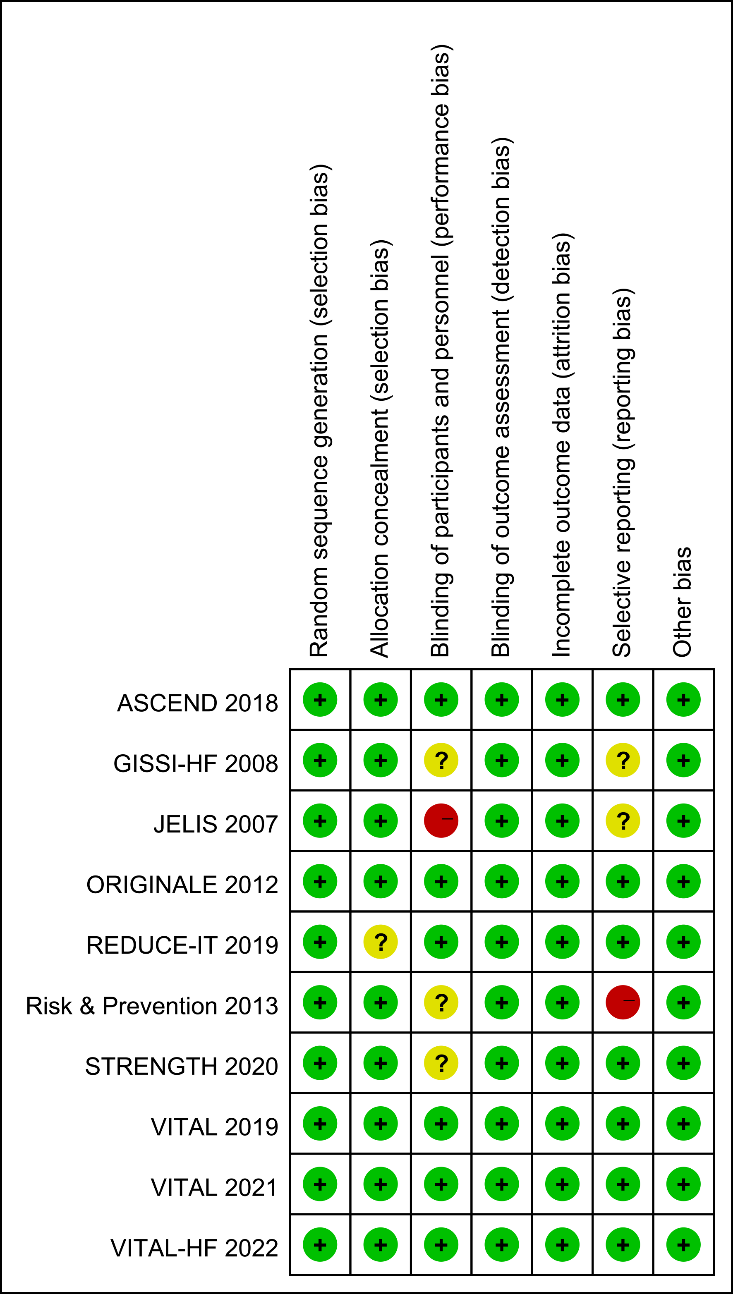


**Figure S1.** Risk of bias figure. Green, low risk; yellow, unclear risk; red, high risk.


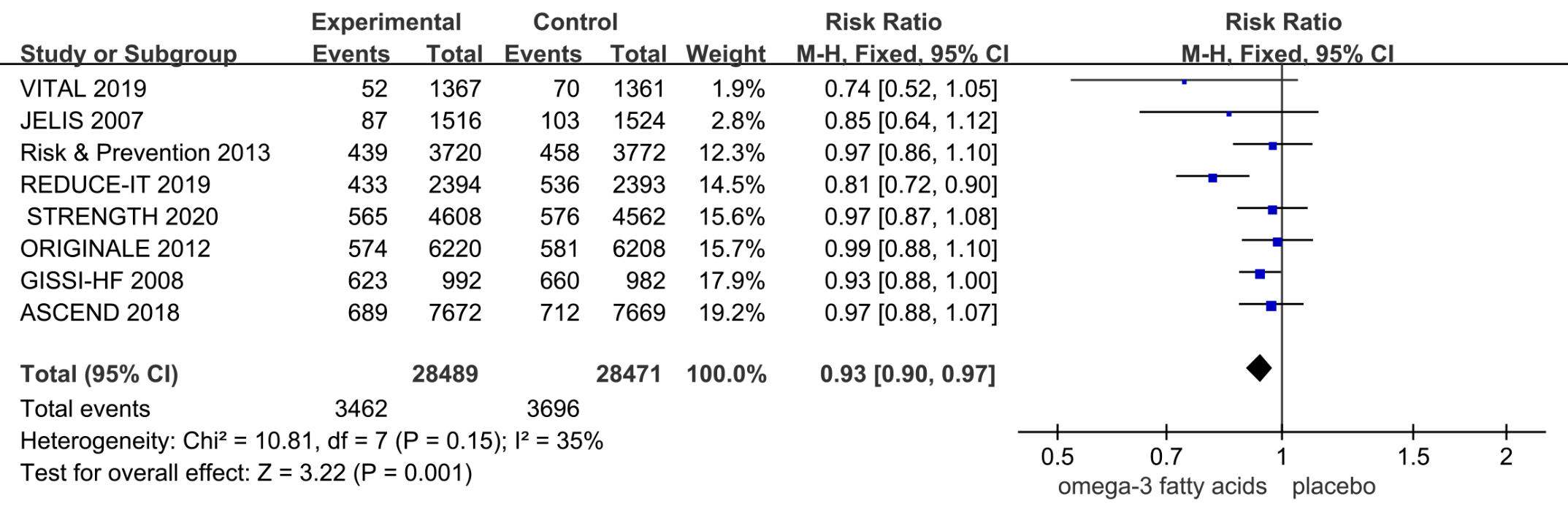


**Figure S2.** Pooled association between omega-3 fatty acid supplementation and risk of cardiovascular disease in participants with diabetes (including VITAL 2019).


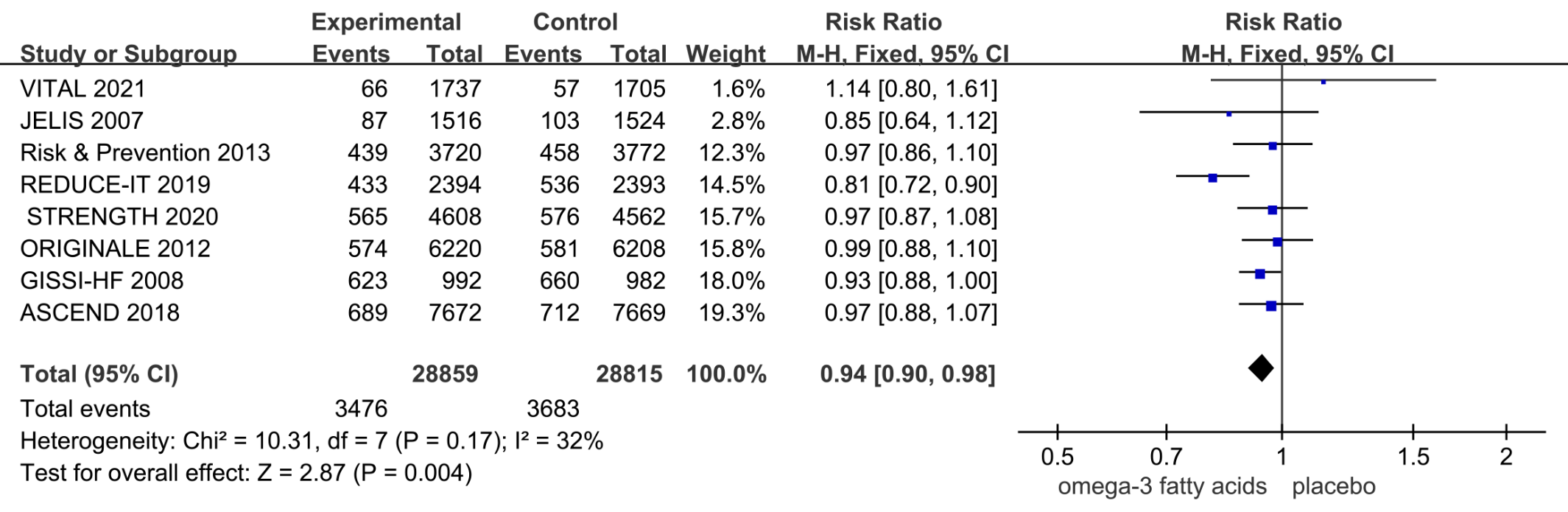


**Figure S3.** Pooled association between omega-3 fatty acid supplementation and risk of cardiovascular disease in participants with diabetes (including VITAL 2021).


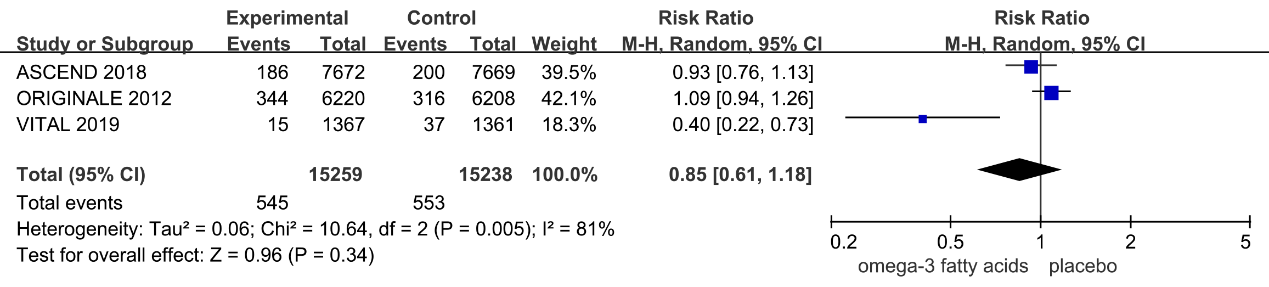


**Figure S4.** Pooled association between omega-3 fatty acid supplementation and risk of total myocardial infarction in participants with diabetes. Total myocardial infarction includes fatal and/or nonfatal myocardial infarction.


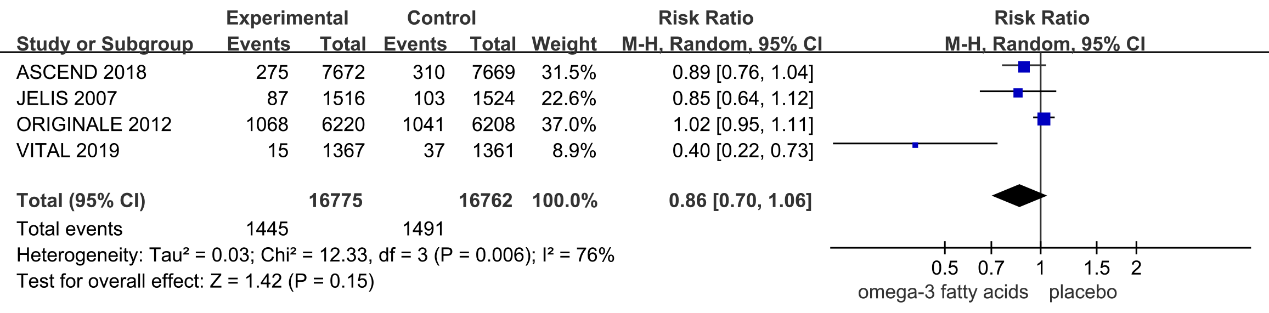


**Figure S5.** Pooled associations between omega-3 fatty acid supplementation and risk of coronary artery disease, including myocardial infarction, angina, coronary death, or coronary revascularization, in participants with diabetes.


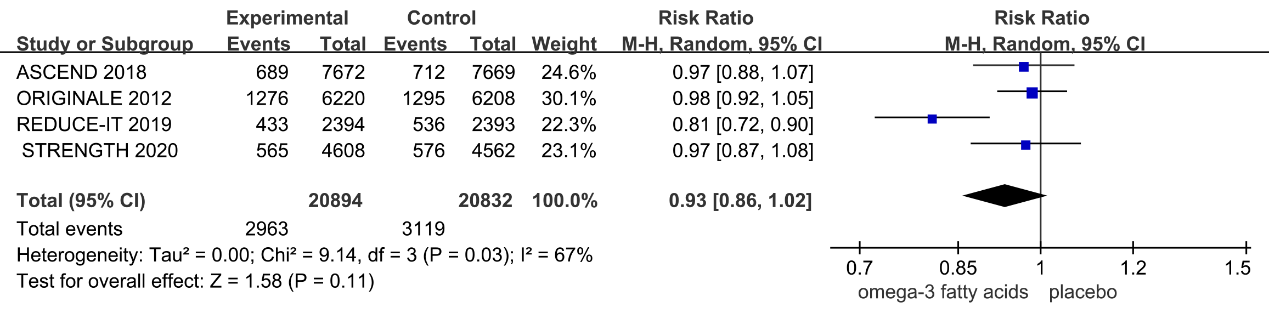


**Figure S6.** Pooled associations between omega-3 fatty acids and risk of major vascular events, including nonfatal myocardial infarction, nonfatal stroke, death from cardiovascular disease, or revascularization, in participants with diabetes.


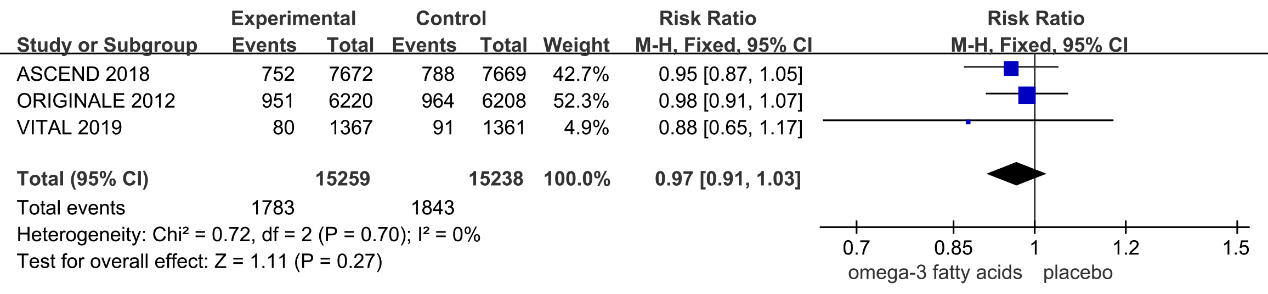


**Figure S7.** Pooled association between omega-3 fatty acid supplementation and risk of total death in participants with diabetes.


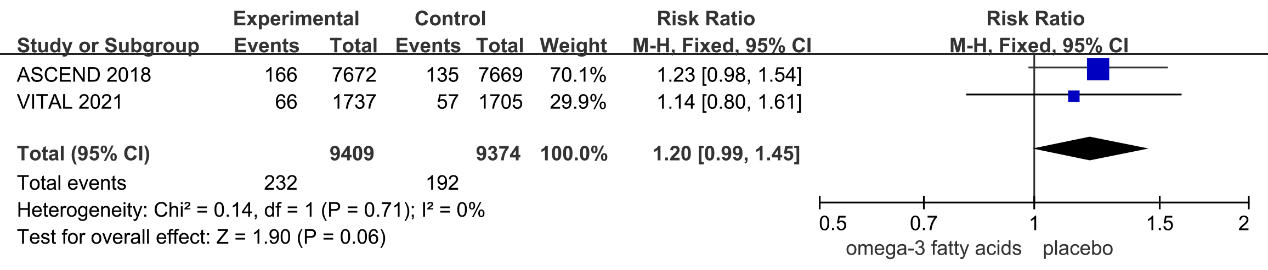


**Figure S8.** Pooled association between omega-3 fatty acid supplementation and risk of atrial fibrillation in participants with diabetes.


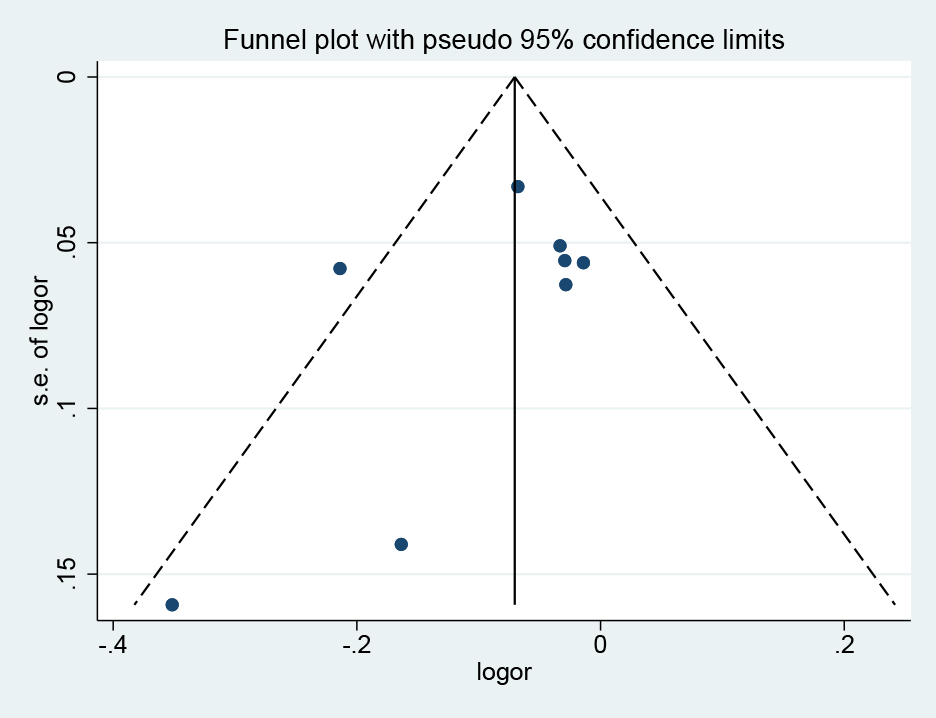


**Figure S9.** Funnel plot of the association between omega-3 fatty acid supplementation and cardiovascular outcomes in diabetes.


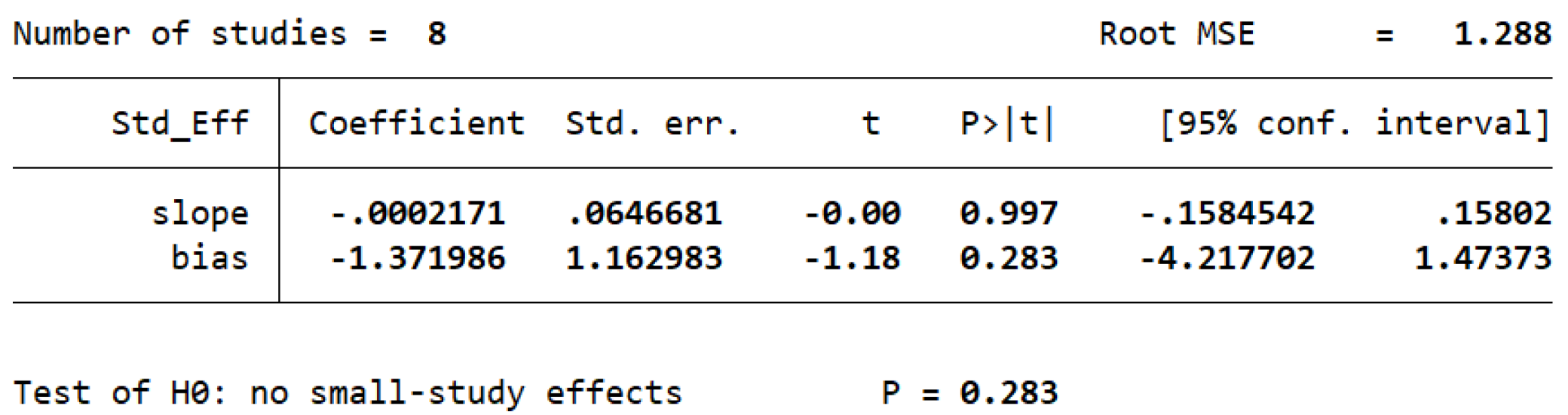


**Figure S10.** Egger’s test of the association between omega-3 fatty acid supplementation and cardiovascular outcomes in diabetes.
